# Pain Management Education for Rural Hospice Family Caregivers: A Pilot Study With Embedded Implementation Evaluation

**DOI:** 10.1177/10499091231191114

**Published:** 2023-07-25

**Authors:** Lauren T. Starr, Karla T. Washington, JoAnn Jabbari, Jacquelyn J. Benson, Debra Parker Oliver, George Demiris, John G. Cagle

**Affiliations:** 1 Department of Biobehavioral Health Sciences, NewCourtland Center for Transitions and Health, 16142 University of Pennsylvania School of Nursing, Philadelphia, PA, USA; 2 12275 Washington University in St Louis School of Medicine, St Louis, MO, USA; 3 499763 Barnes-Jewish College, Goldfarb School of Nursing, St Louis, MO, USA; 4 Department of Biostatistics, Epidemiology and Informatics, University of Pennsylvania Perelman School of Medicine, Philadelphia, PA, USA; 5 Center to Advance Chronic Pain Research, 115980 University of Maryland, School of Social Work, Baltimore, MD, USA

**Keywords:** hospice, family caregiver, pain, education, implementation, rural

## Abstract

**Background:**

Assessing and managing hospice patients’ pain is a common source of anxiety among hospice family caregivers (HFCGs), especially caregivers in rural communities who face special challenges including distance, limited access, and concerns about opioid misuse.

**Objective:**

To pilot test *Ready2Care*, a pain management education intervention for rural HFCGs. We sought to determine whether there was a signal of benefit for clinically-relevant outcomes and to identify contextual factors pertinent to conducting a future randomized clinical trial of *Ready2Care*.

**Methods:**

We conducted a multi-method, single-arm study, enabling completion of paired t-tests comparing pre- and post-intervention measures of caregiver anxiety, pain management self-efficacy, barriers to pain management, and reports of patient pain intensity and corresponding patient and caregiver distress. We concurrently conducted an embedded implementation evaluation via calculation of descriptive statistics (recruitment and retention data) and directed content analysis of brief caregiver interviews.

**Results:**

Twenty-seven (n = 27) HFCGs participated; 15 completed the study. Among completers, significant improvement was observed in patient pain intensity (average 1.4 points decrease on 0-10 scale) and in overall pain experience. No statistically significant changes were detected in caregiver anxiety, barriers to pain management, or pain management self-efficacy. Facilitators to successful conduct of a future clinical trial included high acceptability of *Ready2Care*, driven by its perceived clarity and relevance to caregivers’ concerns. Barriers included lower-than-anticipated accrual and an attrition rate of nearly 44%.

**Conclusion:**

A multisite clinical trial of *Ready2Care* is warranted; however, its success may require more effective recruitment and retention strategies for rural caregiver participants.

## Introduction

Each year, millions of Americans with serious and life-limiting illnesses receive hospice services intended to promote comfort at the end of life.^1^ In the United States, most hospice patients receive care in the community^1^ with family members and friends providing the majority of their care, often with little training, preparation, or support.^2^ Anxiety among these hospice family caregivers (HFCGs) is widespread,^3,4^ with clinically significant prevalence 6 times greater than that of the general population.^5–7^ A common source of anxiety among HFCGs is the challenge of assessing and managing hospice patients’ pain.^8^

HFCGs frequently express discomfort administering pain medications, fearing under- or over-medicating patients.^2,9,10^ They struggle to assess pain^11^ and worry about medication side effects, such as confusion, sedation, and constipation.^2,9,10^ Many report challenges communicating with healthcare providers about pain.^12^ HFCGs who witness uncontrolled pain often feel highly distressed^13^ and are at increased risk for poor long-term outcomes, such as social role impairment, decreased energy, negative perceptions of general health, and major depressive disorder.^14^

HFCGs managing pain in rural communities face unique challenges.^15^ Their homes are often geographically distant from friends, family members, and hospice agencies, limiting access to practical, informational, and emotional support related to pain management.^15–17^ At times, rural cultures emphasize self-reliance and toughness, potentially decreasing the likelihood that rural HFCGs will reach out to others for pain management assistance, further limiting their support.^18^ Furthermore, HFCGs living in rural communities typically have limited access to specialty-trained palliative care professionals, community services (e.g., 24-hour pharmacies), and personal care providers. In addition, social concerns about pain medications (e.g., fear of addiction, stigma associated with use) are particularly pronounced in rural communities, which have been disproportionately affected by opioid misuse.^19,20^ Taken together, these realities underscore the pressing need for evidence-based interventions to support rural HFCGs’ pain management efforts.

*Ready2Care* is a remotely-delivered pain management education intervention for rural HFCGs that is adapted from Cagle and colleagues’ EMPOWER (Effective Management of Pain: Overcoming Worries to Enable Relief) intervention.^21^ EMPOWER has been shown to increase HFCGs’ knowledge about pain management, reduce HFCGs’ concerns about pain and pain medications, and lower patient pain.^21^ *Ready2Care* is delivered in 3 phases: (1) an initial 30 minute telephone screening and provision of tailored educational content based on participant concerns or misperceptions about pain management identified during the screening, (2) mail and/or email delivery of written educational materials (ie, the *Ready2Care* pain education brochure), which participants are asked to review, and (3) a 5-10 minute follow-up call 1 week later to confirm receipt of the written materials, address any new or remaining questions about *Ready2Care* content, and remind participants of ongoing pain management support from their hospice interdisciplinary team. For research purposes, a third 30-minute phone call 1 week later is used to explore participant response to the intervention and any remaining pain management concerns.

The *Ready2Care* educational brochure features common concerns about pain management in hospice care (addiction, tolerance, medication side effects, denying pain, perception of drug-seeking behavior, fear of overmedicating care recipient, not wanting to bother others, and concern treatments will not work) and evidence-based information to address each concern, including education about non-medication adjuvant treatments. The brochure also features information about how to manage medications, including how to stay informed, organized, and how to keep track of medication administration; how to use reminders; when and how to communicate with the hospice team; and hospice team contact information. To support health literacy accessibility, the brochure’s language reads at a 7.4 Flesch-Kinkaid grade level, indicating strong readability, and is presented in easy-to-read font. For the interactive component of *Ready2Care*, nurse interventionists verbally share scripted educational messages in response to identified pain management barriers (eg, addiction concerns, misperceptions about medication side effects), discuss HFCGs’ questions and concerns about these educational messages in the context of their care recipient’s personal needs, and coach HFCGs to discuss their concerns and questions with their interdisciplinary hospice team. In conversation, nurse interventionists show empathy and provide encouragement and affirmation.

### Study Aim

Although randomized clinical trials (RCTs) are recognized as the gold standard for generating evidence on the effectiveness of interventions in hospice care,^22^ they often fail to produce results that can be confidently applied in real-world clinical decision making.^23^ To improve the eventual informativeness of clinical trials, Taylor and Kowalkowski^23^ recommend conducting pre-RCT pilot studies guided by implementation science, enabling identification of facilitators and barriers to the conduct of future RCTs in addition to outcomes appropriate for more definitive investigation in future research. Consistent with this recommendation, our team of researchers and hospice clinicians conducted a pilot study of *Ready2Care,* a pain management education intervention for rural HFCGs. In doing so, we sought to determine whether there was a signal of benefit of *Ready2Care* regarding clinically relevant outcomes and to identify contextual factors pertinent to the conduct of a future randomized clinical trial of the intervention.

## Methods

We conducted a multi-method, single-arm, pre-post pilot study with an embedded implementation evaluation informed by the Consolidated Framework for Implementation Research (CFIR),^24^ Proctor’s conceptual model of implementation research,^25^ and the checklist of goals for pilot studies of psychosocial interventions in palliative care created in collaboration with the Palliative Care Research Cooperative group.^26^ CFIR is an evidence-based implementation framework designed to help researchers identify determinants (i.e., barriers or facilitators) of implementation outcomes, including both clinical outcomes that reflect the impact of an intervention (also referred to as “innovation outcomes”) and outcomes that measure the extent to which an intervention is successfully implemented in clinical settings.^24^ Proctor’s model of implementation research links intervention and implementation strategies with 3 types of outcomes, including implementation outcomes (e.g., feasibility, acceptability), service outcomes (e.g., effectiveness, person-centeredness), and client outcomes (e.g., satisfaction, symptomatology).^25^ Finally, the checklist of goals for pilot studies of psychosocial interventions in palliative care outlines specific objectives a pilot study within the palliative care domain should seek to achieve, such as testing recruitment methods and estimating participant retention.^26^

### Participants and Setting

HFCGs were eligible to participate in the study if they met the following criteria: were (1) age 18 or older, (2) an informal (i.e., unpaid) family caregiver receiving services from 1 of 2 rural hospice sites with which our team had an established research partnership, (3) able to speak and read English, and (4) caring for a hospice patient with a Palliative Performance Scale (PPS) score of 30% or higher (to avoid attempting to recruit family caregivers of patients who were actively dying and to allow sufficient time to deliver the *Ready2Care* intervention prior to hospice patient death). As is customary in hospice, we broadly defined “family caregiver” to include all individuals identified as such in the medical record; legal or biological relationships were not required. For this pilot study, HFCGs of patients with any diagnosis were included.

### Data Collection

Eligible participants were recruited through partnership with a large hospice agency that included 2 sites serving predominantly rural areas of 1 Midwestern state. This agency authorized the research team to access, use, and disclose personal health information solely for the purposes of the study through signing a HIPAA authorization letter. Research staff generated a list of new hospice admissions and located the contact information for individuals listed as newly admitted patients’ family caregiver(s) of record. After confirming that each hospice patient had a PPS score of 30% or greater^27^ and waiting a minimum of 4 days following the hospice admission to ensure regular hospice staff had an opportunity to complete required initial visits and assessments, research staff contacted HFCGs by telephone, briefly explained the study, gauged caregivers’ interest in learning more about the study, provided additional detail for interested parties, and invited potential participants to provide verbal consent to enroll in the study. Recruitment was paused for 1 month during the winter holidays.

Recruitment benchmarks for HFCGs in rural settings were not available due to an absence of published studies in the field. However, our team set recruitment goals based on data obtained from the rural site of a recently concluded HFCG study^21^ that used a recruitment process similar to *Ready2Care*. In that study, research staff were unable to contact 9.5% of 262 eligible referrals from a rural hospice site. Of the remaining 237 HFCGs researchers were able to contact, 28.6% consented, with non-consenting caregivers reporting being “uninterested” and “too busy” as major reasons for declining participation. Based on these benchmark data and a prediction that we would identify an estimated 200 eligible HFCG referrals over the duration of the study, our goal was to consent 50 HFCGs into the *Ready2Care* pilot*.* Finding no published research on rural HFCG interventions of comparable length to *Ready2Care*, we opted not to establish a priori retention goals.

### Design and Measures

In this pilot study, 2 trained researchers delivered the intervention. Both are registered nurses; 1 has a PhD in Nursing and the other is a candidate for a PhD in Nursing Science. During the first of 3 phone calls with study interventionists, HFCGs provided basic demographic data and completed a baseline survey that included a series of items and standardized measures assessing HFCGs’ concerns about pain and pain management (measured with the EMPOWER barriers to pain management scale)^28^; self-efficacy in pain management (measured with selected items from the Modified Caregiver Self-Efficacy in Pain Management scale, CSEPMS)^29^; reports of patient pain intensity and corresponding patient and caregiver distress (measured with the Family Pain Questionnaire 3-item experience subscale^30^ and a single-item caregiver proxy pain rating using a scale from 0 [no pain] to 10 [worst possible pain]); and caregiver anxiety (measured with the Generalized Anxiety Disorder [GAD] 2-item scale).^31^ After baseline data were provided, interventionists reviewed participants’ responses to the EMPOWER scale, verbally relayed scripted educational messages in response to any identified pain management barriers (e.g., concerns or misperceptions), discussed HFCGs’ questions and concerns about these educational messages, and coached participants to address concerns beyond the scope of the intervention with their hospice interdisciplinary team. Following this initial call, interventionists sent a copy of the *Ready2Care* pain education brochure to HFCGs via email and/or U.S. mail, according to HFCG preference.

During a second call approximately 1 week later, interventionists confirmed that HFCGs had received and reviewed the *Ready2Care* pain education brochure and repeated the 3-item Family Pain Questionnaire experience subscale, which assessed caregiver ratings of patient pain intensity and corresponding patient and caregiver distress.

During the third and final call, conducted about 1 week after the second call, interventionists again assessed HFCGs’ concerns about pain and pain management, pain management self-efficacy, reports of patient pain intensity and corresponding patient and caregiver distress, and caregiver anxiety. In addition, consistent with CFIR guidelines,^24^ data on selected contextual factors as potential implementation determinants were collected. This included HFCGs’ access to technology and health literacy (measured with the BRIEF Health Literacy screening tool).^32^ In addition, HFCGs’ perceptions of the intervention were explored via investigator-initiated items and brief qualitative interviews, which were audio-recorded with HFCGs’ consent and later transcribed by a third-party service. At the end of study participation, participants were thanked for their time with up to $50 (by check), depending on how many calls they completed.

The Washington University in St Louis Institutional Review Board approved the study (#202103035). All participants provided verbal consent prior to study enrollment. Participant data were securely stored on protected REDCap servers to maintain strict privacy standards.

### Analysis

Descriptive statistics were used to measure recruitment (absolute number of referrals received, number of referrals received as a percentage of total agency admissions, percentage of referred caregivers who enrolled in the study), retention (percentage of enrolled participants retained in the study through days 7 and 14), and all study measures to provide statistical parameters helpful in planning future research. In addition, comparisons between pre- and post-*Ready2Care* caregiver-reported patient pain ratings, anxiety (2-item GAD) scores, EMPOWER barriers to pain management scores, CSEPMS individual item scores, and Family Pain Questionnaire subscale scores were made using paired t-tests.

A directed content analysis^33^ using NVivo v. 12 was applied to participants’ responses to open-ended questions about perceptions of the *Ready2Care* intervention in the context of researcher observations and field notes. An initial list of predetermined codes, based on the study’s guiding conceptual frameworks and published research on HFCGs’ experiences with pain management,^2,34^ was applied and updated as needed to accommodate newly identified codes. Two researchers (LTS, KTW) independently coded the interviews, then met to compare coding and refine the code list via discussion and, as needed, resolution of conflicts regarding initial coding decisions. The first author then combined the codes to develop themes, which the research team then reviewed.

## Results

### Participant Characteristics

Table 1 describes the study participants who completed the baseline interview (n = 27) and participants who completed the study (n = 15). Participants who completed the study were on average 65 years old, predominantly non-Hispanic (100%), white (93.3%), the spouse or partner of the hospice patient (53.3%), and were retired (60%). Forty-six percent of participants reported an annual household income of $80,000 or more, and 86.7% reported being able to easily access the internet, with 66.7% reporting near-daily or more frequent internet use. Nearly half (46.7%) of HFCGs reported fair or poor health, and only 20% reported very good or excellent health. The mean of participants’ health literacy scores (measured with the BRIEF Health Literacy screening tool)^32^ was 16.9 (standard deviation, SD = 2.6), which falls between marginal (scores of 13-16, which indicate the respondent may need assistance and may struggle with patient and family education materials) and adequate health literacy (scores of 17-20, which indicate the respondent will likely be able to read and comprehend most patient and family education materials).

**Table 1.** Summary of Participant Characteristics.

	Completed Baseline (n = 27)	Completed Study (n = 15)
**Age**		
Mean	61.4 (15.4)	65.3 (14.4)
Median [min, max]	61.6 [19.6, 87.6]	63.8 [27.6, 87.6]
Missing	2 (7%)	1 (6.7%)
**Race**		
White/Caucasian	24 (89%)	14 (93.3%)
Other	3 (11%)	1 (6.7%)
**Ethnicity**		
Not hispanic	27 (100%)	15 (100%)
**Relationship to patient**		
Spouse/Partner	11 (41%)	8 (53.3%)
Child	11 (41%)	4 (26.7%)
Parent	1 (4%)	1 (6.7%)
Sibling	1 (4%)	1 (6.7%)
Other relative	1 (4%)	0 (0%)
Other	2 (7%)	1 (6.7%)
**Highest level of education**		
Elementary/Middle	1 (4%)	1 (6.7%)
Some high school	1 (4%)	1 (6.7%)
High school or GED	8 (30%)	4 (26.7%)
Some college	10 (37%)	7 (46.7%)
Technical/Trade/Associate’s	3 (11%)	1 (6.7%)
College degree	3 (11%)	1 (6.7%)
Graduate degree	1 (4%)	0 (0%)
**Employment status**		
Employed full-time	8 (30%)	3 (20%)
Employed part-time	2 (7%)	0 (0%)
Unemployed	3 (11%)	2 (13.3%)
Retired	13 (48%)	9 (60%)
Other	1 (4%)	1 (6.7%)
**Annual household income**		
Under $20,000	1 (4%)	0 (0%)
$20,000- $39,999	3 (13%)	2 (15%)
$40,000-$59,000	3 (13%)	2 (15%)
$60,000-$79,999	3 (13%)	3 (23%)
$80,000-$99,999	4 (17%)	2 (15%)
Over $100,000	9 (39%)	4 (31%)
Missing	4 (15%)	2 (13.3%)
**Internet access**		
Unable to easily access the internet	5 (19%)	2 (13.3%)
Able to easily access the internet	22 (81%)	13 (86.7%)
**Internet usage in past 2 Weeks**		
Never	4 (15%)	4 (26.7%)
Several days	2 (7%)	0 (0%)
More than half the days	1 (4%)	1 (6.7%)
Nearly every day or more	20 (74%)	10 (66.7%)
**Participant health rating**		
Excellent	4 (15%)	1 (6.7%)
Very good	6 (22%)	2 (13.3%)
Good	9 (33%)	5 (33.3%)
Fair	2 (7%)	1 (6.7%)
Poor	6 (22%)	6 (40%)
**Patient has cancer**		
Yes	14 (54%)	6 (43%)
No	12 (46%)	8 (57%)
Missing	1 (4%)	1 (6.7%)
**Patient has dementia**		
Yes	6 (22%)	4 (26.7%)
No	21 (78%)	11 (73.3%)

Note: Due to rounding, percentages may not total 100%.

### Acceptability

Overall, HFCGs found the *Ready2Care* intervention highly acceptable (Table 2). On a scale of 0 to 10, with 0 representing poor and ten representing excellent, caregivers rated the quality of *Ready2Care*’s written information (i.e., the pain education brochure) 9.1 (mean; standard deviation, SD = 1.6) and rated the quality of their discussions with interventionists about this information 9.7 (mean; SD = .9). Most caregivers (80%) said there was nothing they disliked about the *Ready2Care* pain education brochure and/or recommended researchers keep it the same, while 87% of caregivers said there was nothing they disliked about the intervention’s discussions and/or recommended researchers keep the discussions the same.

**Table 2.** Summary of Ready2Care Quality Ratings.

	Overall (n = 15)
**Quality of written information (0-10 rating)**	
Mean (SD)	9.1 (1.6)
Median [min, max]	10 [5, 10]
Missing	1 (6.7%)
**Quality of telephone discussion (0-10 rating)**	
Mean (SD)	9.7 (.9)
Median [min, max]	10 [7, 10]
Missing	0 (.0%)

Table 3 provides specific examples of *Ready2Care* features HFCGs reported liking. Caregivers reported liking that the intervention was “informative,” “clear,” engaging, and designed at what they deemed an appropriate literacy level. Caregivers liked how *Ready2Care* normalized pain experiences near end of life; increased caregiver confidence in hospice pain management; provided socio-emotional support and a caring, empathic response; provided real-time answers to caregiver questions; and included non-pharmacologic ways to manage pain. Caregivers also reported liking the format of the written brochure. Regarding intervention delivery, 81% of caregivers on the final call reported not wanting to experience the intervention exclusively online, indicating the importance of in-person or telephone-based human interaction.

**Table 3.** Insights About Implementation of the Ready2Care Intervention Among Rural Hospice Family Caregivers.

Theme	Quotation	Caregiver
**What Caregivers Liked About Ready2Care**		
Informative, helpful	“It answered all the main questions of the concerns I had.”	107
	“I think it was very informative. There’s a lot that we never considered, especially when it comes to pain management.”	069
Engaging	“I thought it was very clear. It helped me think about some of things I hadn’t thought about before.”	040
	“You didn’t make me fall asleep.”	078
Appropriate health literacy level	“Didn’t have any really big words in it.”	056
	“You have it laid out really simple and I think keep it like that because my sister could read it, understand it, and she’s not as educated as far as high school and college as I am, so it was easy to understand from both standpoints—my sister and myself […]it was the simplicity, but you learn a lot. It’s very appreciated when you’re overwhelmed with information.”	105
Increases caregiver confidence in pain management	“It made me feel like I was doing okay, that I wasn’t making mistakes. I Think that’s 1 of the biggest worries: That people are going to make [mistakes], you worry about making a mistake. I Really liked their stressing the part that I didn’t have to worry about what I was giving him, and I didn’t have to worry about giving him too much, and I could get more. Those are the things that I think—people having questions in their mind, “Am I doing this right? Or should I save some?”	091
Normalizes pain experiences at end-of-life	“The fact that there’s been so many people going through it that whatever they tell you is pretty much true.”	059
Informed caregivers of nonverbal communication of pain through behavior	“I would say it was very informational because I had no clue about it and it explained to me perfectly so that I knew what to look for as far as the grimacing that I’d—yeah, I learned a lot from that.”	105
	“In the picture, I, first, I want to tell you that it really helped me because of the positioning of the mouth. Because I recognized that instantly when I saw it [before I even] read anything, and the wrinkles around the eyes. The mouth’s shape and everything, I was like, ‘Oh, boy. That is it.’ You know?”	091 (care-recipient has vascular dementia)
Provides socio-emotional support	“[My daughter] thought it was very good that you were calling and going through all this with me. I Think it’s very good, too, because I haven’t had anybody that I could really talk with that seems to [understand].”	040
	“I’ve had a lot of people contacting me. It just helps to be able to talk about things.”	090
Provides real-time answers to questions	“I told them how nice it was to have somebody that asked me questions and I could get answers and you guys understood. I Really did like that.”	079
Provides a caring, empathic response	“The fact that you care… Just knowing that somebody’s there for you.”	059
Included non-pharmacologic ways to manage pain	“Well, the thing about [using not] just pain medicine, [but also using] aromatherapy.”	107
Effective format of the written brochure	“The layout was great.”	105
	“I like how it’s broken down into different common concerns.”	041
**Caregivers’ recommended changes to improve Ready2Care**		
Personalize the intervention	“Then, as far as what you talk about, I think the personalization of each case [would be helpful], as opposed to, ‘this is brain cancer. This is what it does,’ so […] you could add a little bit more of that personalization.”	091
Add more information	“I think maybe it might go into a little bit more detail. I’m not sure because everybody is so different. I don’t know if that’s even possible, like I said, with constipation, giving up all these things, because maybe other people aren’t having these same problems.”	105
Adapt for dementia and other conditions with cognitive impairment	“I mentioned the picture [of the grimacing face] because—I keep stressing that—because it was a relief thing to me. It was like a light bulb went off. She said, ‘Oh, yes. I’ve seen that on Grandpa.’ You know?”	091 (care-recipient has vascular dementia)
Revise brochure language in terms of recommended actions, not pain management concerns	“[The brochure’s language] needs to be turned around some way to be, ‘Don’t worry about addiction,’ or something like that, because people take away with the bullet points. That’s all they’re going to see.”	041

Table 3 also describes *Ready2Care* components caregivers recommended changing. A minority of caregivers recommended *Ready2Care* personalize content to a patient’s specific illness and symptoms (e.g., how to administer pain medication to a person with continuous hiccups). Caregivers of persons with dementia or cognitive impairment strongly valued the intervention, recommending researchers adapt *Ready2Care* to their specific informational needs, given limited understanding of non-verbal pain behaviors and assessing pain in this population. Similarly, a minority of caregivers reported wanting the intervention to include more detail (e.g., additional and/or more specific strategies to address constipation). Finally, a minority of caregivers recommended small changes to the brochure itself. For example, 1 caregiver recommended researchers revise the brochure language such that pain “concerns” are instead presented as recommended actions caregivers can take, and that the hospice telephone number be included on all pages for easy access.

### Feasibility

**Figure 1.**
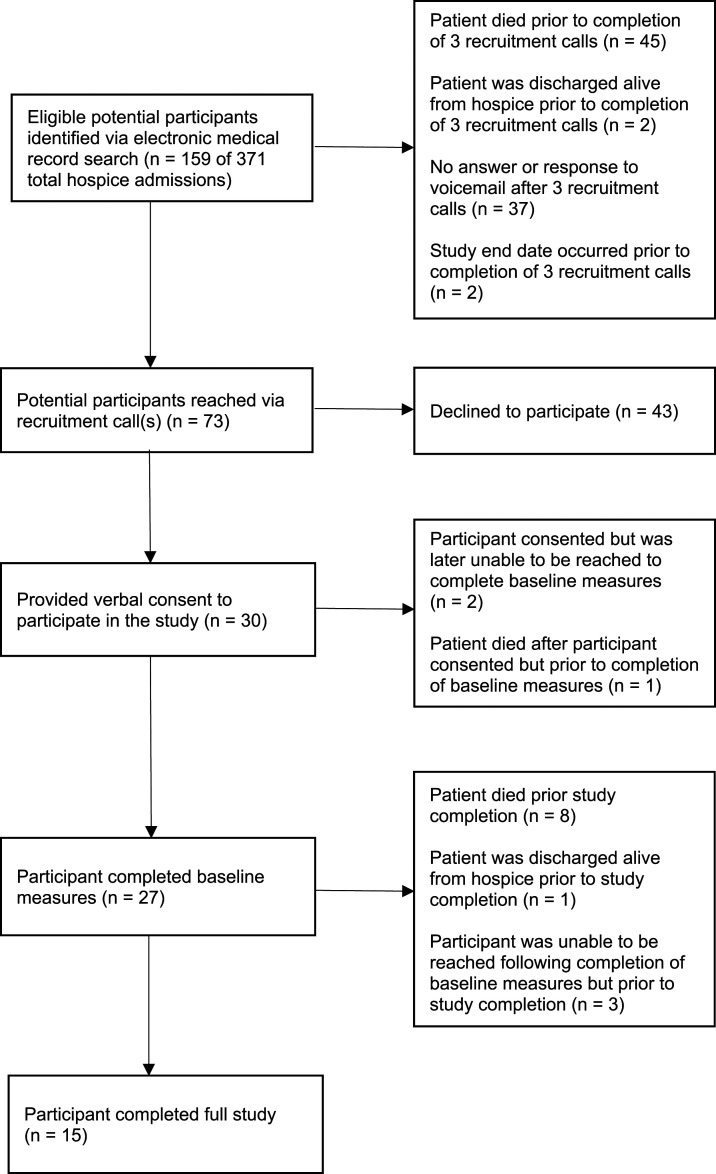
*Ready2Care* family caregiver participant recruitment and retention.

Feasibility results were mixed. The feasibility of delivering *Ready2Care* to participants once enrolled in the study was supported, as we were able to conduct initial screenings and discuss tailored educational messages with 27 of the 30 HFCGs who consented. However, we identified numerous challenges associated with the feasibility of recruiting and retaining a sufficiently large sample of rural HFCGs to support future, large-scale testing of the intervention. Ultimately, we fell short of our recruitment goal of 50 HFCGs, and only half of the HFCGs who consented to study participation completed the full 14-day study protocol. Figure 1 presents the flow diagram of participant recruitment, enrollment, and study completion.

#### Recruitment

From September 7, 2021 to August 1, 2022, we identified 159 eligible referrals, which represented 42.9% of agency hospice admissions during this period. Of these referrals, 37 caregivers did not answer any of the interventionists’ 3 phone calls or respond to interventionist voicemails (23.3% of referrals), with 45 hospice patients dying prior to completion of the 3 recruitment calls (28.3%). Thirty participants provided verbal consent to participate in the study (18.9%), but only 27 caregivers completed the baseline assessment (17.0% of referrals).

#### Retention

Of 27 caregivers who completed baseline interviews, 19 were retained through day 7 (11.9% of referrals, 70.4% of participants who completed baseline interviews) and 15 caregivers completed the study (9.4% of referrals, 55.6% of participants who completed baseline interviews). Of the 19 caregivers who completed day 7, 14 (73.7%) had reviewed the *Ready2Care* brochure and 5 (26.3%) had not. Reasons caregivers gave for having not yet accessed the brochure by day 7 included lack of time or lack of access to mail.

### *Ready2 Care* Outcomes

We were able to detect a signal of potential benefit of *Ready2Care* regarding 2 clinically relevant outcomes. Caregivers who completed the study (n = 15) reported a statistically significant improvement in patient pain from baseline (4.7 average pain rating, 2.7 SD) to post-intervention (3.3 average pain rating, 2.6 SD) (P = .01) (Table 4). The intervention also demonstrated significant improvement in caregiver perceptions of patient pain and pain-related distress (FPQ 3-item pain experience subscale), with an average score of 19.4 (5.6 SD) at baseline compared to 14.6 (8.1 SD) post-intervention (P = .02). Differences in caregiver concerns about pain management (EMPOWER barriers to pain management), caregiver self-efficacy in pain management (CSEPMS), and caregiver anxiety (GAD-2) did not statistically differ pre- and post-intervention.

**Table 4.** Full Study Participant Variables at Baseline and Post-Intervention for Ready2Care.

	Baseline (n = 15)	Post-intervention (n = 15)	P	
**Caregiver rating of Patient’s current pain level (range 0-10)**				
Mean (SD)	4.7 (2.7)	3.3 (2.6)		**.012**
Median [min, max]	4 [0, 10]	4 [0, 9]		
**EMPOWER pain barriers (range 0-80)**				
Mean (SD)	18.5 (14.0)	18.9 (12.0)		.70
Median [min, max]	15 [2, 49]	15 [4, 46]		
Missing	2 (13.3%)	0 (0%)		
**Caregiver self-efficacy in pain management (CSEPMS) single items (range 0-10)**				
*Manage pain during daily activities*				
Mean (SD)	6.6 (2.6)	7.8 (2.2)		.05
Missing	0 (0%)	1 (6.6%)		
*Cope with mild-to-moderate pain*				
Mean (SD)	7.2 (2.5)	8.1 (2.0)		.26
Missing	2 (13.3%)	3 (20%)		
*Cope with severe pain*				
Mean (SD)	6.3 (2.5)	7.0 (2.8)		.32
Missing	4 (26.7%)	5 (33.3%)		
*Decrease pain quite a bit*				
Mean (SD)	6.6 (2.6)	7.2 (2.8)		.31
Missing	2 (13.3%)	2 (13.3%)		
*Keep pain from interfering with sleep*				
Mean (SD)	5.9 (3.1)	6.6 (3.0)		.56
Missing	2 (13.3%)	1 (6.7%)		
*Make a small-to-moderate reduction in pain, no extra medication*				
Mean (SD)	4.2 (3.8)	5.7 (3.1)		.07
Missing	3 (20%)	3 (20%)		
*Make a large-to-moderate reduction in pain, no extra medication*				
Mean (SD)	2.5 (3.0)	3.8 (3.4)		.13
Missing	4 (26.7%)	4 (26.7%)		
**Family pain questionnaire (FPQ) 3-item experience subscale (range 0-30)**				
Mean (SD)	19.4 (5.56)	14.6 (8.1)		**.016**
Median [min, max]	18 [11, 29]	14.5 [0, 28]		
Missing	1 (6.7%)	1 (6.7%)		
**Generalized anxiety disorder 2-item (GAD-2) (range 0-6)**				
Mean (SD)	4.0 (1.8)	4.4 (1.6)		.39
Median [min, max]	4 [0, 6]	5 [2, 6]		

P-values for test of mean difference. Statistically significant P-values <.05 are indicated in bold.

SD, standard deviation; EMPOWER, Effective Management of Pain: Overcoming Worries to Enable Relief.

The CSEPMS is scored so that higher values are more desirable, whereas the other variables are scored so that lower scores are more desirable.

Table 5 qualitatively describes perceptions of *Ready2Care*’s impact*,* as reported by HFCGs in interviews. Caregivers reported improved understanding of how pain management works with hospice patients, increased attention to signs of pain in the patient, improved understanding of pain medication tolerance and dosing over time, reduced fears of opioid pain medicine addiction, increased comfort giving opioid pain medication, improved pain among patients, affirmed understanding of pain management practices, improved caregiver communication through documentation, and increased collaboration with the hospice team. They also noted that the intervention enabled caregivers to take action, reduced fears and misconceptions about hospice, improved caregiver feelings of support, and improved caregiver coping. Finally, caregivers reported that *Ready2Care* increased or improved communication about pain management with other caregivers: 66.7% reported that they shared the *Ready2Care* pain management brochure and/or discussion information with others.

**Table 5.** Caregiver-Reported Outcomes of the *Ready2Care* Pain Education Intervention.

Outcomes	Caregiver Quotations	Caregiver
Improved caregiver understanding of how pain management works	“I don’t think either 1 of us had a clear understanding of how [pain management] worked, and I think we do now. I think, just with all of the negative connotations with people who had abused things in the past, it’s just a completely different situation. […] I think separating ourselves from what we thought [pain management] was compared to what it actually is for us and for us was very helpful.”	069
Increased attention to signs of patient pain	“I will definitely keep a closer eye on [patient] as far as watching when he moves around or gets up or this and that, to make sure and ask him if he is having pain or anxiety. If I see it, I’ll know it’s happening, and I can take care of it.”	135
Improved caregiver understanding of pain medication side effects	“I understand it better now and I realize that even though the pain meds could [affect] the confusion, the confusion’s going to be there anyway and I can take away the pain.”	105
Improved caregiver understanding of pain medication tolerance and dosing over time	“I’m [now] aware that […] when things get worse […] there will be heavier medication that you can take.”	107
Reduced caregiver fears of opioid addiction	“I [was] always […] very wary of giving [patient] any type of an opioid, or anything […] in the past. I Thought she could become addicted. This is a different situation where, in the past, she wasn’t even having pain, but now she is. It’s all about keeping her comfortable.”	069
Increased caregiver comfort giving opioid pain medications	“I was thinking that when you first started talking to me about the pain management I was a little more wary and a little more … uncomfortable giving the pain medication just because she’s never had to be on it, other than tylenol or the normal over-the-counter stuff. I Guess in my mind, knowing the situation that she’s in now, you know, I am a lot more comfortable with it.”	069
Contributed to improving patient pain	[Before *Ready2Care*,] “I really wasn’t sure about whether he was in pain. I Was giving him 1 hydrocodone in the morning and then, a lot of times, he would tell me he didn’t need it—he was okay. He just wanted to go to bed, so I tried to get away with that. Now, not so much. Because of talking to you and [hospice nurse] and you and the triage nurse, everybody, [he] has been very, very good.”	091
Affirmed understanding or practices related to pain management	“I think [the hospice team] had all given me a little bit of—what do you want to call it?—a start in understanding some of it. Then that brochure just brought it all to mind, ‘Okay. I’m thinking right.’”	091
Improved inter-caregiver communication via improved documentation of pain medication administration	“Using reminders [was helpful] because we have 3 to 4 caregivers for my dad, just based off work and stuff. We write down on a sheet and put what his medication is for the day and what times he needs it at, and then just every day they check it off as they’re giving the medication.”	133
Increased collaboration with hospice team	“I really wasn’t sure [if he was in pain or not]. I Think talking to you and everybody and then the triage nurse has made me feel a little bit more confident and suggested that maybe I needed to give a little bit more pain medicine than I had been doing before.”	091
Increased communication about pain management with other caregivers via sharing of information obtained through the intervention	“[I shared with my husband] mainly the pain management [content]. I Explained to him on how it works, and he completely understood. He’s on the same page.”	069
Reduced caregiver fears or misconceptions about hospice	“My daughter was afraid hospice was going to come in and kill her grandma. I was afraid of the same thing. That’s why I told them I didn’t want it. I told [daughter] that you called, and you discussed it with me, and I understand it better, and that’s why I allowed it to come in. You helped my mom out quite a bit. You may not know this, but you have. […] I really didn’t know what was happening until you talked to me and made me feel better about it.”	078
Improved caregiver feelings of support	“I told [my friends] how nice it was to have somebody that asked me questions and I could get answers and you guys understood. I Really did like that.”	078
Improved caregiver ability to cope	“I’m going to be able to handle it here with help that I’m getting.”	091
Enabled caregiver to take action	“It gave me direction to take it other than, ‘hey, what are we going to do about this and this?’ [or] ‘hey, I learned this about this—' You know?”	105

## Discussion

In this pilot study of an educational pain management intervention for rural hospice family caregivers, we found *Ready2Care* to be highly acceptable and feasible to implement clinically. Quantitative study results underscored the clinical promise of *Ready2Care* as a strategy to improve pain and related distress, while qualitative findings illustrated *Ready2Care*’s potential to improve pain management understanding and confidence among caregivers, facilitate action to relive pain among caregivers, improve pain-related communication across caregivers, and increase collaboration with the hospice team.

Research-oriented feasibility measures, however, highlighted pronounced difficulties recruiting and retaining HFCGs from rural hospice programs for research purposes. Our findings are consistent with other studies that indicate inherent challenges with recruitment and attrition in hospice research, typically due to the limited interest or availability of caregivers, short hospice enrollment periods, and patient death.^35,36^ More research is needed to understand optimal recruitment and retention methods for HFCG studies. Some HFCG researchers recruit participants after hospice agencies have pre-screened participants and presented intervention programs to participants during nurse or social worker home visits.^35,37^ This pre-screening referral process is significantly more resource and time-intensive than our team’s recruitment strategy yet may result in higher consent rates. For example, 1 recent study of an intervention for HFCGs that used this recruitment method yielded 47 eligible referrals from 2 non-rural hospice sites during a 9-month period; 43% consented to participate, 38% started the intervention, and 21% completed the intervention (n = 10).^37^

Study findings also shed light on numerous contextual factors relevant to the conduct of a future randomized clinical trial of *Ready2Care*. Providing care for an individual nearing the end of life is often burdensome and can be both physically and emotionally exhausting.^38^ This may be particularly true for caregivers who are themselves in fair or poor health, which was the case for nearly half of the participants who completed the *Ready2Care* intervention in this study. Overwhelmed family caregivers may be disinclined to participate in research or, if they consent to participate, may have insufficient time to engage in research-related activities (e.g., reviewing the *Ready2Care* pain education brochure) outside of scheduled encounters with the research team. Future *Ready2Care* studies should be designed with the busy lives of family caregivers in mind. Recruitment and retention challenges should be anticipated and accounted for in planning efforts (e.g., conducting power analyses, deciding upon the number of sites to engage), and required activities between intervention sessions should be minimized if not eliminated entirely. Difficulties with recruitment of hospice family caregivers in research is well documented.^35,36^ Hospice enrollment typically occurs late in the illness trajectory, often within the last 2 weeks of life, limiting the time frame researchers can conduct studies. Out of respect for families, we waited 4 days after hospice enrollment before contacting caregivers, further reducing our window of opportunity to engage HFCGs. HFCGs provide difficult hands-on care at an emotionally stressful and demanding time, reducing participant receptivity and challenging informed consent.^35,36^ Recruitment of hospice caregivers in rural settings is particularly challenging given lower adoption of hospice in rural areas^39^ and the typically small size of rural hospice programs.

Early in the research process, we realized our recruitment goals were too high due to fewer than expected eligible referrals and a significantly higher non-response rate (23.3% non-response in our study compared to 9.5% in another recently completed clinical trial including similar participants). We attribute this difference to the difficulty of caregiving and, possibly, the phone numbers from which interventionists called participants, which did not match local area codes and may have been dismissed as suspected telemarketing. Future research will require more hospice agency partnerships and rural sites and use of phones with local numbers to decrease automatic refusal. In addition, the consent rate (18.9%) of *Ready2Care* was lower than the study we used to set recruitment goals (28.6%), which may be due to differences in the intervention requirements and perceived benefits. HFCG studies that use hospice staff to recruit participants demonstrate higher conversion rates^35^ but also increase adoption barriers.

Difficulties retaining HFCG participants due to hospice patient death further compounded sample size issues.^35,36^ In *Ready2Care*, 90% of participants who verbally consented to participate completed the baseline interview, 63.3% were retained through day 7, and 50% completed the study. Based on other studies, hospice patient death and lack of time were likely contributing factors to attrition.^35,36^ Future research should account for high attrition and identify ways to increase retention among rural participants.

More specific findings regarding the acceptability of *Ready2Care* focused on its remote delivery. In both quantitative and qualitative reports, caregivers expressed strong positive sentiments and appreciation for the intervention as delivered via telephone. This finding is consistent with a recent systematic review that found telehealth interventions, specifically those conducted over the telephone like *Ready2Care*, to be effective at developing caregiving skills, improving psychological functioning among family caregivers of persons with chronic illnesses, and increasing collaboration.^40^ As found in previous research, the experience of the interventionists, 2 registered nurses, and use of a structured tool to guide care practice positively contributed to discussing pain management concerns and best practices.^41,42^

Study participants also emphasized their appreciation for the socio-emotional support provided by *Ready2Care* interventionists, whom they described as caring and empathic, with some expressing sentiments similar to the 1 shared by a HFCG who explained that *Ready2Care* was “very good” because they had not previously “had anybody [they] could really talk with that seems to [understand].” Prior research has found loneliness or “a sense of profound isolation” to be an important contextual factor in the end-of-life caregiving experience,^43^ suggesting that future iterations of *Ready2Care* will be best received and perhaps most likely to be effective if meaningful contact with interventionists is retained. That is not to suggest that additional technologies could not play a role in research with rural HFCGs. Web-based delivery of participant surveys, for example, appears to have a high likelihood of feasibility, as nearly 87% of study participants reported being able to easily access the internet. However, human contact in the delivery of the intervention itself appears to be an important feature to continue.

Participants’ expressed desires for personal interaction and feeling understood and their appreciation of *Ready2Care*’s support reflect a deep need for programs that address the social and emotional needs of HFCGs. Social support, which has multiple dimensions (emotional, appraisal/esteem, companionship, informational, and instructional^44^) is a critical factor in improving caregiver mental and physical health and reducing caregiver burden.^44–47^ Unfortunately, as patients near the end-of-life, social support for caregivers from friends and family often decreases,^48^ making support for HFCGs especially important. While it is unknown which social support dimensions *Ready2Care* addressed, expansion of social support features of *Ready2Care* may be warranted. In addition, clinicians should consider developing social support groups for HFCGs. Rural HFCGs unable to join in-person support groups due to distance or responsibilities may benefit from online support groups. One recent RCT found that hospice family caregivers who participated in a private online support group on Facebook experienced statistically significant decreases in depression compared with HFCGs in the usual care control group.^21^

Study participants also indicated researchers should consider adapting *Ready2Care* to conditions commonly encountered in hospice, such as dementia and other illnesses that may result in cognitive impairment, as pain may be under-controlled in these populations due to caregiver difficulties assessing and managing pain among patients who are non-verbal or who experience other communication challenges,^49^ highlighting the unique disease-specific contexts in which hospice caregiving takes place. Importantly, however, study findings suggest that any future changes to the *Ready2Care* pain education brochure should be made in such a way that its clarity and ease of understanding are retained, particularly given that the average of participants’ health literacy scores fell between cut-offs for “marginal” and “adequate” levels, and numerous participants commented positively about the lower literacy level at which the Ready2Care pain education brochure is currently written.

Although HFCGs reported numerous positive outcomes and actions they attributed to participation in *Ready2Care*, some variables (e.g., caregiver anxiety, caregiver pain management self-efficacy) did not improve in a statistically detectable manner. While this finding may be due to small sample size, interventionist field notes suggest that for some caregivers, notably those who did not initially think their family member experienced pain, concerns about pain and their management of pain may have increased as the patient began experiencing new pain or as HFCGs became more aware of pain through improved assessment promoted by *Ready2Care*. Similarly, evidence suggests that family caregivers experience greater anxiety as a patient approaches death,^50^ which may explain why caregiver anxiety did not improve post-intervention. Additional research involving larger samples and more rigorous study design features (e.g., participant randomization, blinding of outcome assessors) are needed to more fully capture the impact of *Ready2Care* and to better understand how it affects dimensions of HFCGs’ emotional experiences.

## Conclusion

*Ready2Care*, a remotely-delivered adaptation of Cagle and colleagues’ educational pain management intervention, EMPOWER, was shown to be highly acceptable among HFCGs in rural settings and innovative in its effectiveness at improving hospice pain management outcomes. Future research should explore better recruitment and retention methods to improve feasibility and should consider adaptations to common hospice conditions, such as dementia, in which pain is often poorly understood and under-treated. A multisite clinical trial of *Ready2Care* is recommended.
